# Psychological impacts of exercise snacks vs. traditional exercise: a comparative study

**DOI:** 10.3389/fpsyg.2026.1917848

**Published:** 2026-10-08

**Authors:** Sheng Yan, Yantong Sun, Zhengquan Zhang, Zongdi Zhao, Linjun Liu

**Affiliations:** 1 Department of Physical and Aesthetic Education, Wuhan Technical University, Wuhan, China; 2 Institute of Sports Technologies and Physical Education, Moscow State University of Sport and Tourism, Moscow, Russia; 3 Faculty of Pharmacy, Lincoln University College, Petaling Jaya, Malaysia

**Keywords:** behavioral adherence, cognitive load, decision fatigue, exercise snacks, psychometric profile, traditional exercise

## Abstract

This study conducted a 4-week randomized controlled trial to compare the psychometric profiles and adherence patterns of Exercise Snacks Group (ESG)—a high-frequency, fragmented exercise protocol—against Traditional Exercise Group (TEG)—continuous exercise—under volume-equated conditions (90 min/week at 40% $\dot{V}O_{2max}$). A total of 180 participants were evaluated on Perceived Stress (PS), Decision Fatigue and Cognitive Load (DFCL), and Physical Activity Enjoyment (PAE; reverse-scored to reflect negative psychological appraisal). The results indicated that the ESG cohort experienced significantly higher levels of PS, DFCL, and lower physical activity enjoyment [higher PAE inverse score, alongside a markedly higher attrition rate (15.56%) compared to the TEG cohort (5.56%).] Intra-group correlation analyses confirmed operational independence among psychometric indicators across both modalities, demonstrating that high-frequency protocols introduce distinct cognitive and affective costs without confounding measurement structures. These findings indicate that while ESG offers a schedule-flexible approach for physical health, its high-frequency demands introduce notable “behavioral friction” and cognitive strain. Consequently, practitioners and researchers must carefully balance the metabolic benefits of fragmented exercise against its psychological costs to ensure sustainable adherence for general populations.

## 1 Introduction

In the contemporary landscape of lifestyle medicine, the challenge of maintaining physical activity amidst increasingly demanding and time-constrained daily schedules has become a critical public health concern. Traditional exercise models, which emphasize long, continuous bouts of physical exertion, often struggle to accommodate the logistical constraints of modern life. In response, the concept of fragmented exercise—short, high-frequency, repeated bouts of activity—has emerged as a scientifically viable paradigm to bridge the gap between sedentary lifestyles and established health recommendations. There is a growing consensus within the exercise physiology community that intermittent physical activity, when appropriately dosed, provides substantial metabolic and cardiovascular benefits comparable to, or in some cases superior to, traditional continuous exercise protocols.

Extensive research has consistently highlighted the physiological efficacy of this fragmented approach (Yin et al., 2024, 2023; Jones et al., 2024; Weston et al., 2025). Studies have demonstrated that distributed exercise sessions effectively improve cardiorespiratory fitness, enhance glycemic control, and mitigate the long-term risks of metabolic syndrome (Islam et al., 2022; Francois et al., 2014). By reducing the threshold for participation and eliminating the need for prolonged time blocks, fragmented exercise protocols have shown a remarkable capacity to increase the volume of movement in previously sedentary populations. Furthermore, the physiological adaptations induced by these repeated, acute bouts of exertion—such as enhanced mitochondrial biogenesis and improved vascular endothelial function—underscore the robust biological potency of this approach, positioning it as an efficient strategy to counteract the adverse consequences of prolonged sedentary behavior.

However, despite the well-documented physiological benefits, the psychological and cognitive landscape of fragmented exercise remains inadequately mapped. It is important to acknowledge a terminological and conceptual nuance regarding protocol design: while classical “exercise snacks” are frequently defined in prior literature as ultra-short (e.g., ≤ 1–2 min) bouts spread multiple times throughout a single day (Jones et al., 2024; Babir et al., 2025), emerging lifestyle interventions often extend this framework into slightly longer, discrete distributed blocks (e.g., 10–15 min per session) to fit practical work-break scheduling (Yin et al., 2024; Fyfe et al., 2022). In this study, we operationalize the Exercise Snacks Group (ESG) as a 15-min high-frequency protocol; while expanding beyond the ultra-short micro-bout definition, it preserves the core fragmentation and task-switching characteristics that challenge daily routines. The high-frequency nature of these protocols introduces a complex behavioral dimension: constant task-switching, continuous schedule monitoring, and repetitive engagement. It remains unclear whether the convenience of fragmented exercise is offset by an accumulation of mental load or “behavioral friction” that may paradoxically impact long-term adherence.

To address this critical gap, the present study conducts a 4-week randomized controlled trial comparing a high-frequency fragmented exercise protocol (Exercise Snacks Group, ESG) with a volume-equated traditional continuous exercise protocol (Traditional Exercise Group, TEG). Drawing upon Lazarus and Folkman's Transactional Model of Stress and Coping, Sweller's Cognitive Load Theory (Cohen et al., 1983; Sweller, 1988; Kendzierski and DeCarlo, 1991), and Self-Determination Theory, we tested the following directional hypotheses: (1) due to the constant micro-scheduling and task-switching friction, the ESG protocol will induce significantly higher levels of Perceived Stress (PS) and Decision Fatigue and Cognitive Load (DFCL) compared to the TEG model; (2) the repetitive daily burden will alter Physical Activity Enjoyment (PAE) profiles; and (3) the accumulation of behavioral friction in the high-frequency ESG paradigm will translate into a substantially higher attrition and replacement rate.

## 2 Results

### 2.1 Chronological flow and procedural architecture of the protocol

As illustrated in Figure 1, the experimental intervention was executed over a 4-week longitudinal schedule, strictly adhering to the established procedural architecture. All participants successfully completed the baseline psychometric assessments and were subsequently randomized into either the Exercise Snacks Group (ESG, *n* = 90) or the Traditional Exercise Group (TEG, *n* = 90).

**Figure 1:**
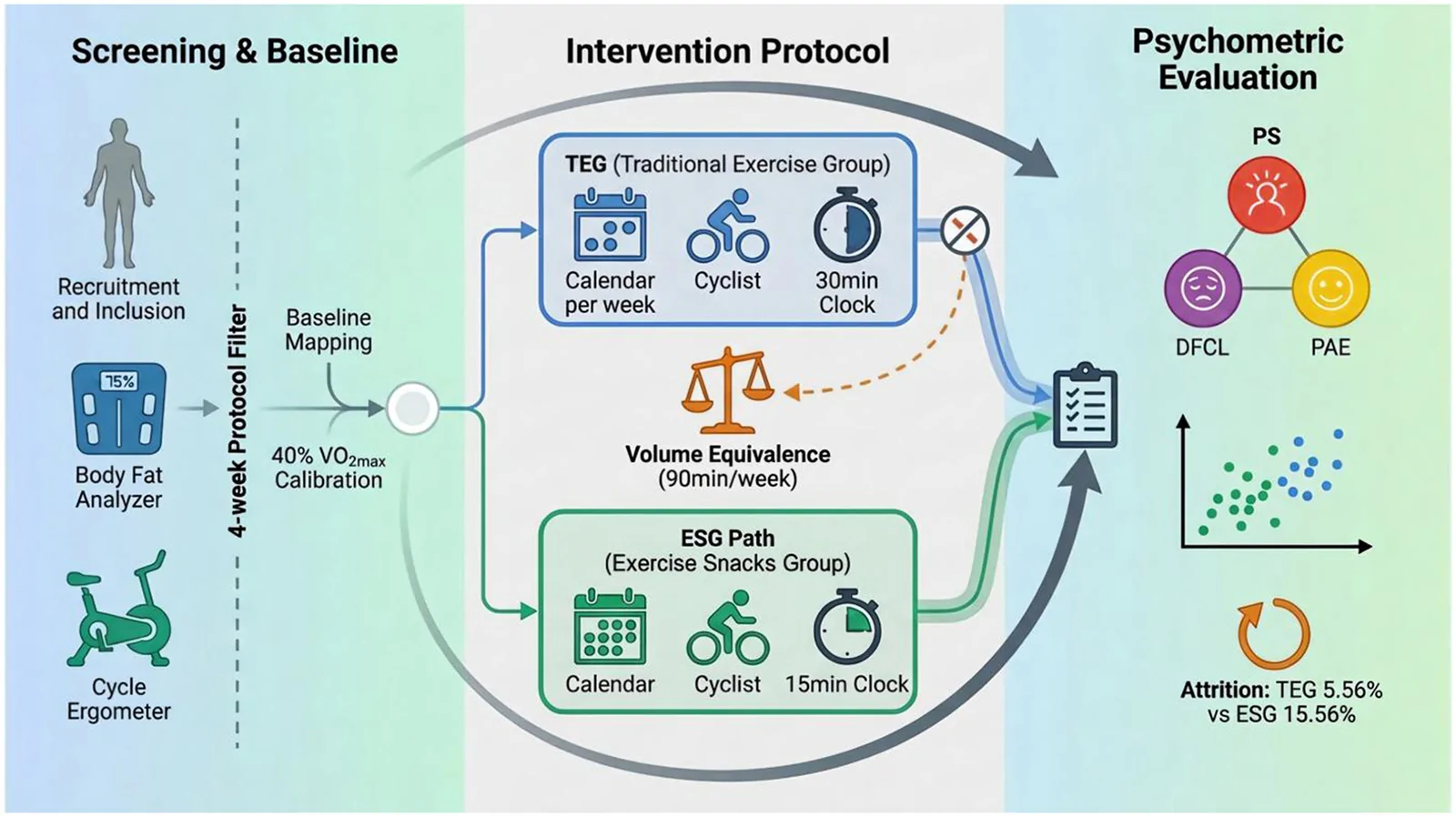
Chronological flow and procedural architecture of the 4-week experimental intervention.

The intervention protocol was standardized across all subjects, with continuous monitoring ensuring compliance with the prescribed training modalities. Following the 4-week duration, post-intervention data collection was conducted under controlled conditions to minimize external interference. The operational flow—from initial allocation to the final psychometric evaluation—was maintained consistently, providing a stable empirical foundation for the subsequent analysis of psychometric outcomes.

### 2.2 Post-intervention outcomes of target psychometric indicators

As illustrated in Table 1, a comparative analysis was executed to evaluate the numerical profiles of the three designated psychometric indicators following the 4-week intervention paradigm. Using the Traditional Exercise Group (TEG) as the reference baseline, the behavioral and psychological changes demonstrated by the Exercise Snacks Group (ESG) revealed distinct divergence gradients across different dimensions.

**Table 1:** Statistical overview of the target psychometric indicators across different groups.

Indicator	Group (*M* ±SD)		Partial η^2^	Cohen's *f*
	ESG (*n* = 90)	TEG (*n* = 90)		
Perceived stress	33.80 ± 6.15	24.50 ± 5.20	0.403	0.821
Decision fatigue and cognitive load	22.60 ± 4.64	18.40 ± 4.24	0.184	0.475
Physical activity enjoyment	32.80 ± 5.21	20.50 ± 4.46	0.620	1.276

ESG, Exercise Snacks Group; TEG, Traditional Exercise Group.

#### 2.2.1 Identifiable variations and relative metrics closeness

Among the evaluated dimensions, the smallest numerical gap between the two intervention modalities was captured in Decision Fatigue and Cognitive Load (DFCL). Compared to the TEG baseline (18.40 ± 4.24), the ESG post-test score rose moderately to 22.60 ± 4.64. This relative closeness indicates that while the high-frequency interruption of the ESG protocol demands a slightly higher cognitive task-switching cost, it does not induce an exponential or severe accumulation of cognitive exhaustion compared to traditional continuous training.

#### 2.2.2 Medium-to-large discrepancy gradients

A more pronounced variance was identified in Perceived Stress (PS). The ESG exhibited a substantial numerical increase to 33.80 ± 6.15 against the TEG baseline score of 24.50 ± 5.20. This notable divergence demonstrates that the 4-week high-frequency intervention markedly shifts individual perceptions of time urgency and environmental load, creating a distinct stress profile compared to the conventional continuous model.

#### 2.2.3 Maximum discrepancy topology

The most substantial gap between the two training models emerged in Physical Activity Enjoyment (PAE). The ESG mean score surged to 32.80 ± 5.21, showing an immense numerical distance from the TEG baseline of 20.50 ± 4.46. The descriptive analysis for PAE revealed a dominant gap, establishing it as the most differentiated metric in the study.

### 2.3 Distribution profiles of post-intervention psychometric outcomes

As illustrated in Figure 2, the distribution patterns of the three psychometric indicators were observed for the Exercise Snacks Group (ESG) and the Traditional Exercise Group (TEG) following the 4-week intervention. The scatter plots delineate the individual score distribution and density for each cohort, providing a comprehensive overview of the data spread across the evaluated domains. The visual representation indicates that while there are expected overlaps in the score ranges between the two groups, the central tendencies and distribution clusters differ distinctly across the intervention modalities. To facilitate direct comparison, the plots reveal that the ESG cohort's score density consistently shifts toward the higher-value regions of the scale in Perceived Stress (PS) and Physical Activity Enjoyment (PAE), whereas the TEG cohort's scores remain more tightly clustered within the lower-to-middle range. This clear visual separation in distribution density allows for an immediate identification of the divergence in psychometric outcomes.

**Figure 2:**
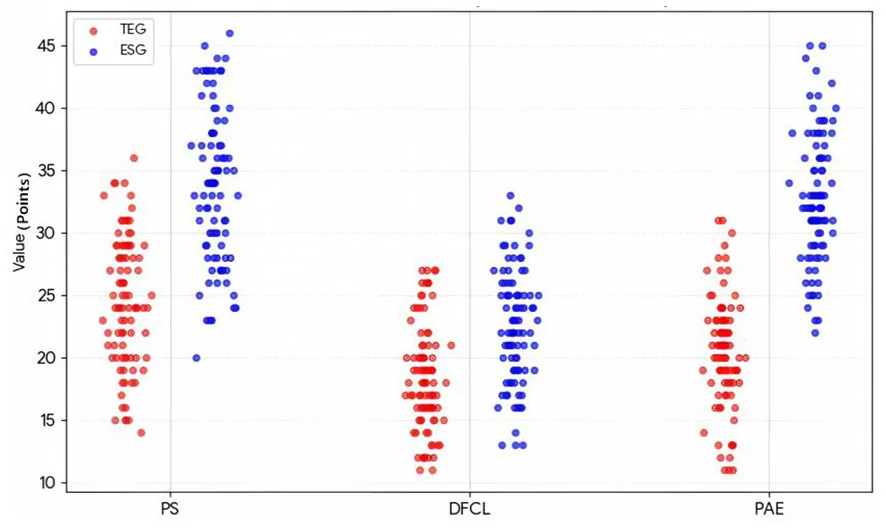
Distribution of psychometric outcomes across the Exercise Snacks Group (ESG) and the Traditional Exercise Group (TEG).

### 2.4 Comparative observation of intra-group correlation characteristics

When examining the correlations within each specific group, a visible difference emerged between the two training modalities.

Within the TEG cohort, a weak but statistically significant positive correlation was observed between perceived stress and decision fatigue (*r* = 0.218, *p* < 0.05). This statistical covariance aligns with general behavioral observations where cumulative lifestyle strain typically co-varies with cognitive exhaustion under standard, undisturbed daily routines.

In contrast, this specific linear relationship was not observed within the ESG cohort, where the correlation coefficient between perceived stress and decision fatigue neared zero (*r* = −0.030, *p*>0.05). The lack of a linear correlation within the experimental group suggests that the high-frequency intervention interacts differently with the baseline relationship between stress and cognitive load. These descriptive variations indicate that while “Exercise Snacks” relate to notable shifts in global psychometric outcomes, they also demonstrate a different internal correlation structure compared to the traditional continuous exercise model.

### 2.5 Attrition and standby replacement rates

During the 4-week intervention period, participant attrition due to an inability to maintain long-term adherence was tracked and addressed via the pre-established standby protocol. In the Traditional Exercise Group (TEG), five participants withdrew from the study because they felt unable to sustain the routine; these slots were immediately refilled by randomly selecting five replacements from the standby back-up pool, thereby maintaining the final sample at exactly 90 participants. Conversely, a higher behavioral resistance was observed in the Exercise Snacks Group (ESG), where 14 participants withdrew prematurely due to their inability to adhere to the high-frequency protocol; these 14 vacancies were subsequently filled by randomly drawing 14 new volunteers from the standby back-up pool, ensuring that the final analyzed cohort also remained at exactly 90 participants.

The clear difference in dropout rates between the two conditions provides useful insight into the real-world viability of these exercise habits. Based on the final cohort of 90 participants per group, the dropout proportion in the TEG was 5.56%, whereas the ESG required a 15.56% replacement rate. While this numeric difference points to a higher discontinuation rate in the fragmented protocol, attributing these withdrawals exclusively to psychological disruption requires cautious interpretation, as attrition is often driven by a combination of logistical constraints, scheduling conflicts, and individual preferences. Nonetheless, these descriptive percentages suggest that high-frequency exercise routines may encounter distinct adherence challenges in real-world settings (Goodman and Blum, 1996) .

## 3 Methods

### 3.1 Experimental protocol and operational timeline

Table 2 systematically details the operational progression and chronological framework of this study. As charted in this timeline, the empirical investigation spanned three sequential phases, ensuring a standardized experimental flow.

**Table 2:** Operational framework and phased design of the experimental protocol.

Phase	Target cohort and Key metrics
Preliminary screening	Body fat percentage screening
	$\dot{V}{\text{O}}_{2\text{max}}$ baseline mapping
Formal intervention (weeks 1–4)	Traditional Exercise Group (TEG)^a^ (*n* = 90)
	Exercise Snacks Group (ESG)^b^ (*n* = 90)
Psychometric evaluation (post-intervention)	Perceived stress scale
	Decision fatigue and cognitive load scale
	Physical activity enjoyment scale

^a^TEG: three sessions per week, 30 min per session continuous cycling at 40% $\dot{V}{\text{O}}_{2\text{max}}$.^b^ESG: six sessions per week, 15 min per session distributed cycling at 40% $\dot{V}{\text{O}}_{2\text{max}}$.

### 3.2 Participants and selection criteria

#### 3.2.1 Participant recruitment and dynamic flow

A total of 199 healthy young adults were initially recruited and screened for this 4-week exercise intervention.

To eliminate allocation bias while maintaining a strictly balanced design, participants were assigned to either the Traditional Exercise Group (TEG) or the Exercise Snacks Group (ESG) using a computer-generated block randomization method.

Furthermore, a formalized waiting-list pool was established during the baseline recruitment phase, consisting of eligible volunteers who agreed to be placed on standby (Schulz et al., 2010; Sheffet et al., 2015). In the event that an initially assigned participant exercised their ethical right to withdraw from either group without justification during this window, the dynamic replacement protocol was immediately triggered: a replacement was randomly selected directly from the standby back-up pool using a random number generator and seamlessly mobilized to take over the slot. This dual-layer methodological control guaranteed a robust, mathematically balanced *N* = 90 per group for the final post-intervention analysis.

#### 3.2.2 Inclusion and exclusion criteria

To optimize the compliance of the cohorts and ensure the convenience of recruitment, the target population was specifically restricted to university students aged between 18 and 27 years. This demographic was chosen because the high-frequency intervention protocol (especially for the Exercise Snacks group) required flexible schedule adjustments, which could pose severe logistical challenges and conflict with the rigid, non-negotiable schedules of full-time corporate employees.

To minimize the confounding effects of extreme physical composition on perceived exercise exertion and potential baseline physical discomfort, participants were required to fall within a normal body fat percentage range (12%–25% for males and 18%–33% for females), which was screened at baseline using a body composition analyzer (Model SH-V9, Shuhua Sports Co., Ltd., China). This specific normative range was adopted based on the established classification guidelines of the American College of Sports Medicine (ACSM), which defines these thresholds as the standard for satisfactory health and low risk for metabolic or musculoskeletal complications in young adults, thereby effectively excluding individuals with clinical obesity or severe underweight status who might experience disproportionate physical distress during exertion. Additionally, candidates were required to demonstrate the physical capacity to perform light-to-moderate intensity aerobic exercises and functional movements, including but not limited to brisk walking, stationary cycling, or basic bodyweight training, without any medical restrictions, while demonstrating the behavioral willingness to adhere to the assigned exercise regimen for four consecutive weeks.

Conversely, candidates were excluded from participation if they presented any pre-existing medical conditions, acute musculoskeletal injuries, or cardiopulmonary pathological histories that contraindicate physical exertion, as determined by the Physical Activity Readiness Questionnaire (PAR-Q). Furthermore, individuals were excluded if they had concurrent enrollment in professional athletic training, varsity sports programs, or any external structured weight-loss or fitness interventions during the 4-week period, or if they had a documented clinical diagnosis of severe psychiatric disorders or ongoing pharmacological treatments for mental health conditions that would impair their capacity to independently relative operational independence the weekly psychological survey matrices.

### 3.3 Preliminary testing protocol

#### 3.3.1 Body composition assessment

To ensure accurate participant eligibility screening, the baseline body composition assessment was uniformly scheduled at 8:00 a.m. following an overnight fast. This strict control of timing and metabolic state was methodologically necessary to minimize the confounding effects of diurnal fluid shifts and biological clock variables on total body water distribution (Borga et al., 2018; Duren et al., 2008). Because the bioelectrical impedance analysis (BIA) technology utilized by the analyzer depends entirely on tissue conductivity, any recent dietary or fluid consumption could distort the impedance signals, artificially modifying tissue resistance and leading to a misclassification of a candidate's body fat percentage. Conducting the screening at a fixed early morning hour under fasting conditions effectively neutralized the volatile impacts of digestion and daytime hydration cycles. This rigorous approach guaranteed that each candidate's body fat percentage was evaluated under highly standardized conditions, preventing measurement bias from leading to the erroneous inclusion or exclusion of participants during the recruitment phase.

#### 3.3.2 Cardiorespiratory exercise testing

During the preliminary phase, participants completed a ramp incremental cardiorespiratory exercise test (CPET) utilizing an electronically braked cycle ergometer (Model T300, Magene Technology Co., Ltd., Qingdao, China). Throughout the testing procedure, metabolic and respiratory gas exchange metrics were monitored continuously via a breath-by-breath cart system (Model Cardiopulmonary Function Testing System Max-1, Mepun Medical Instrument Co., Ltd., Chengdu, China). The evaluation began with a 3-min baseline warm-up maintaining a steady power output of 50 W. Immediately following this phase, the work rate expanded linearly at a ramp increment of 20 W/min, during which participants were required to maintain a consistent pedaling cadence between 60 and 65 rpm. To elicit maximum cardiopulmonary performance, standardized verbal encouragement was delivered by trained researchers during the final stages of the test. The exercise advanced until participants reached absolute volitional exhaustion and could no longer sustain the designated pedaling speed (Glaab and Taube, 2022; Mezzani, 2017).

The primary physiological marker extracted from this protocol was relative peak oxygen consumption ($\dot{V}{\text{O}}_{2}\text{max\_ml\_kg\_min}$), which represents the maximum volume of oxygen consumed per kilogram of body weight per minute at the point of exhaustion, mathematically defined as:


$$\dot{V}{\text{O}}_{2}\text{max\_ml\_kg\_min}=\frac{\text{Absolute Peak}\dot{V}{\text{O}}_{2}\text{(mL/min)}}{\text{Body Mass (kg)}}$$

The primary purpose of administering this preliminary CPET protocol was to establish an objective, personalized baseline to guarantee prescription precision throughout the subsequent 4-week formal intervention phase. By identifying each individual's exact maximal aerobic capacity, the investigators mathematically quantified a customized target workload calculated at exactly 40% of their unique $\dot{V}{\text{O}}_{2\text{max}}$. This specific 40% threshold was selected in alignment with the American College of Sports Medicine (ACSM) guidelines, which classify this intensity at the lower boundary of moderate-intensity aerobic exercise (Oliveira and Brito, 2024; Garber et al., 2011; Swain and Franklin, 2002; Hansen et al., 1984; Knox-Brown et al., 2025; Raghuveer et al., 2020). Grounded in behavior change theories, this low-to-moderate intensity serves as a strategic physiological baseline for sedentary or recreationally active young adults; it is sufficiently robust to elicit metabolic responses without inducing excessive physical fatigue or perceived exertion, thereby maximizing long-term adherence and preventing baseline physical discomfort from biasing the behavioral outcomes. Consequently, this tailored threshold functioned as the uniform physiological anchor for every intervention session across the study duration. Deploying this individualized calibration ensures that whether a participant was allocated to the high-frequency Exercise Snacks Group (ESG) or the continuous Traditional Exercise Group (TEG), the relative metabolic strain remained rigidly standardized and homogeneous across all intervention sessions, thereby neutralizing any confounding variance that baseline physical fitness might exert on the subsequent behavioral and psychological outcomes.

#### 3.3.3 Individual profiles of baseline VO2max data

To provide a transparent overview of the cardiorespiratory capacity within this study, the individual $\dot{V}{\text{O}}_{2\text{max}}$ values captured during the initial testing are displayed in Figure 3. This graphical representation functions solely to illustrate the baseline distribution, range, and general aerobic fitness level of the entire participant cohort, offering a direct visual reference of the baseline maximum oxygen consumption within the sampled population.

**Figure 3:**
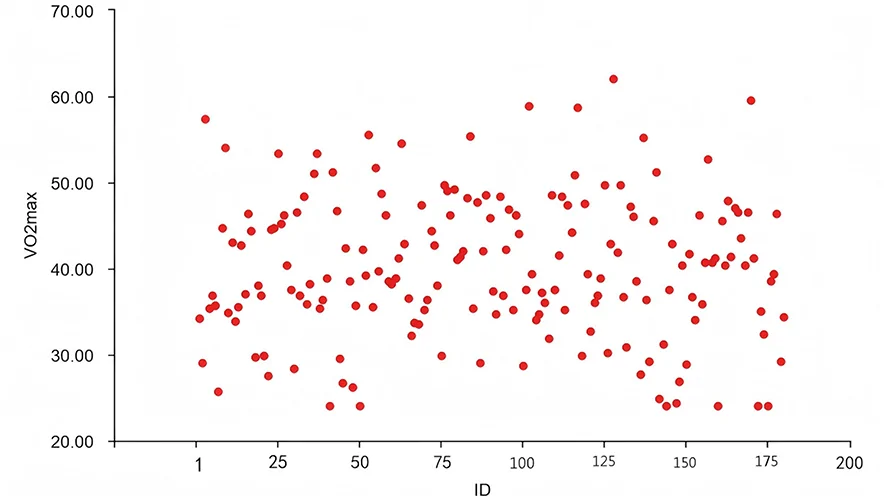
Baseline distribution of maximal aerobic capacity across the entire participant cohort.

In the subsequent intervention phase, the exercise intensity for both the ESG and TEG cohorts was targeted at 40% of their individual maximal aerobic capacity. Participants were instructed to actively self-regulate their pedaling intensity throughout each session, utilizing real-time feedback from the electronically braked cycle ergometers to maintain their exertion as close to this 40% threshold as possible. To account for the physiological fluctuations and inherent equipment response variances, an operational target range of 35%–45% was established, providing a pragmatic buffer for participants to manage during dynamic cycling. This interval is consistent with the American College of Sports Medicine (ACSM) guidelines for moderate-intensity aerobic exercise and ensures that while participants maintained autonomous control, the relative metabolic strain remained within a standardized threshold for both groups. By monitoring these performance metrics consistently, we effectively neutralized potential confounding variances in intensity between the fragmented and continuous exercise protocols.

### 3.4 Formal experimental protocol

#### 3.4.1 Experimental grouping and intervention design

Following the preliminary screening, the 4-week formal intervention was executed under supervised laboratory conditions (Wicker et al., 2015; Nouchi et al., 2014). Participants were required to maintain their habitual dietary intake and physical activity patterns outside the laboratory throughout the study duration.

The two parallel cohorts underwent distinct cycling protocols tailored to manipulate session frequency and duration while equating total weekly exercise volume and intensity.

Traditional Exercise Group (TEG): participants assigned to the TEG performed structured, continuous cycling sessions three times per week, fixed on Mondays, Wednesdays, and Fridays. Each session lasted 30 min continuously, yielding a total weekly exercise duration of 90 min.

Exercise Snacks Group (ESG): participants assigned to the ESG executed shorter, higher-frequency distributed cycling sessions performed six times per week from Monday to Saturday. Each discrete session was restricted to 15 min, also accumulating a total weekly exercise duration of 90 min.

For both experimental groups, the exercise intensity was prescribed at exactly 40% of their predetermined baseline $\dot{V}{\text{O}}_{2\text{max}}$. All exercise bouts were conducted on electronically braked cycle ergometers, with the target workload (in Watts) continuously monitored via real-time power output tracking to ensure precise protocol adherence.

#### 3.4.2 Rationale for total exercise volume selection

The selection of a cumulative weekly exercise duration of 90 min was grounded in a multi-dimensional optimization of physiological efficacy, behavior change theory, and injury prevention tailored to this specific cohort.

Guideline Alignment and Metabolic Threshold: while international guidelines (such as those from the World Health Organization) generally recommend 150 min of moderate-intensity physical activity per week for healthy adults, empirical evidence suggests that in previously sedentary or recreationally active populations, a sub-maximal volume of 90 min per week is a critical threshold. This volume is sufficiently robust to elicit initial cardiorespiratory and metabolic adaptations without overwhelming the participants' physiological baseline (WHO Organization, 2020).

Behavioral Feasibility and Micro-Incentives: from a behavioral psychology perspective, a weekly target of 90 min provides an accessible, low-barrier entry point for habit formation. When fragmented into the ESG protocol, a 15-min session functions as a quintessential “exercise snack”—short enough to eliminate the perceived barrier of “lack of time” and bypass the psychological resistance associated with prolonged exertion, thereby enhancing compliance.

Injury Mitigation and Musculoskeletal Safety: from the participant's safety perspective, non-athletic young adults face significantly elevated risks of overuse injuries, joint strain, and severe delayed onset muscle soreness (DOMS) when abruptly initiating high-volume training. Limiting the weekly volume to 90 min on a low-impact cycle ergometer minimizes mechanical stress on the lower extremity joints, effectively mitigating injury risks and preventing early dropouts driven by physical discomfort.

#### 3.4.3 Attrition replacement and protocol completion

To mitigate experimental attrition and maintain a balanced sample size of *N* = 90 per group, any participant who withdrew was immediately replaced by an individual randomly drawn from the standby volunteer pool. To guarantee methodological uniformity, all replacement participants were strictly required to distinct operational profiles the identical, full 4-week intervention from their respective entry points prior to post-testing.

### 3.5 Post-intervention psychometric evaluation

Exactly within 24 h following the final exercise session of the 4-week protocol, all participants from both the TEG and ESG cohorts underwent a comprehensive psychometric evaluation. This temporal window was strictly enforced to capture the immediate, psychological consolidation of the 4-week intervention while minimizing any confounding recall decay or external weekly stressors.

To guarantee data integrity and participant confidentiality in accordance with institutional ethical standards, the assessment was administered via physical, paper-based questionnaires instead of digital platforms. All participants completed the instruments anonymously, utilizing only the unique, non-identifiable alphanumeric identification codes (IDs) assigned to them during the preliminary screening phase. No personally identifiable information (PII), such as names or student registration numbers, was collected on the survey sheets, ensuring that the subsequent data digitization and statistical analysis remained entirely double-blinded.

To maintain measurement uniformity, all evaluation dimensions derived from the Post-Intervention Questionnaire were structured into a standardized 10-item format scored on an identical 5-point Likert scale, ranging from 1 (Never) to 5 (Very Often). Consequently, each instrument yielded a cumulative score range of 10–50, where higher scores represented greater severities of the respective psychological constructs (or higher levels of negative appraisal regarding physical activity in the case of the enjoyment metric).

#### 3.5.1 Perceived stress scale

The first dimension, Routine Interruption and Uncontrollability, focused on the immediate perceived schedule friction and loss of personal autonomy associated with the physical intervention, which was operationalized via the initial two sequential items of the scale. Specifically, the first item quantified localized schedule disruption by evaluating how often participants felt upset because of unexpected changes in their daily schedules, such as their routine being interrupted by the exercise intervention. This was immediately followed by the second item, which provided a broader appraisal of personal agency by assessing the perceived frequency of experiencing a frustrating lack of control over the important things in their lives over the past month.

The second dimension, Affective Strain and Overload, comprehensively quantified persistent negative emotional states, lifestyle strain, and cumulative cognitive exhaustion spanning the subsequent five items. This segment opened with the third item, which measured generalized psychological distress by tracking how frequently participants felt generally nervous, stressed, or “on edge. ” The fourth and fifth items systematically quantified coping capacity and role overload under the study's protocol, capturing how often participants felt overwhelmed by the challenge of balancing their work or studies with their daily personal responsibilities, alongside whether they felt that the difficulties and demands accumulating in their lives were too heavy for them to handle, respectively. Furthermore, the sixth item targeted the participants' capacity for psychological recovery, evaluating how often they found it difficult to mentally unwind or relax even when they had free time, while the seventh item concluded this dimension by tracking chronic emotional depletion, measuring how often they felt easily irritated by minor hassles in their daily routine.

The third dimension, Time Urgency and Bandwidth Depletion, targeted the acute temporal constraints and psychological resource scarcity surrounding the intervention through the final three items of the instrument. This segment began with the eighth item, which captured the subjective acceleration of time by quantifying how often participants experienced a persistent sense of time urgency, feeling that they were constantly racing against the clock. Next, the ninth item evaluated productivity-related frustration, explicitly tracking how often they felt angry or frustrated because they felt inefficient or unproductive due to frequent daily interruptions. Finally, the sub-scale concluded with the 10th item, which captured global adaptive resilience by evaluating how often participants felt that they lacked the mental bandwidth or social support needed to cope with their daily stressors.

#### 3.5.2 Decision fatigue and cognitive load scale

The first dimension, Task-Switching and Dual-Task Demands, targeted the initial cognitive expenditure and attentional friction required to integrate the exercise protocol into existing academic structures, which was operationalized via the 11th and 12th items of the questionnaire. Specifically, the 11th item quantified the baseline logistical and planning burden by evaluating how often participants felt that planning and scheduling their daily activities, including fitting in their exercise sessions, required significant mental effort. This was immediately complemented by the twelfth item, which captured the specific cognitive fatigue induced by rapid behavioral transitions, measuring how frequently they felt mentally exhausted from constantly shifting their focus back and forth between their work or studies and their exercise routine.

The second dimension, Self-Regulatory Depletion, focused on the erosion of finite volitional resources over the diurnal cycle, spanning the 13th and 14th items. This segment began with the 13th item, which tracked the early behavioral signs of mental exhaustion by assessing how often participants found themselves delaying or procrastinating on making simple decisions because they felt mentally drained. Following this, the 14th item directly quantified the phenomenon of ego depletion by measuring how often they felt that their willpower was completely drained by the end of the day due to the constant need to self-regulate and stick to their schedule.

The third dimension, Cognitive Overload and Choice Evasion, measured the consequences of information saturation and the subsequent tactical retreat from executive functioning through the 15th, 16th, and 19th items. The 15th item initiated this appraisal by tracking micro-logistical saturation, evaluating how often participants felt that their brain was “overloaded” with too many small details and tasks to keep track of throughout the day. This resource depletion was tied to a behavioral avoidance strategy in the 16th item, which measured how frequently they felt a strong desire to avoid making choices altogether, preferring to let others decide or let things slide. The 19th item concluded this dimension by capturing the narrowing of cognitive scope, quantifying how often they felt that their mental bandwidth was so restricted that they could only focus on immediate deadlines, losing sight of the bigger picture.

The fourth dimension, Executive Dysregulation and Mental Fog, comprehensive evaluated the manifest impairments in cognitive clarity and self-control resulting from sustained mental fatigue, through the final three items of the scale. This segment opened with the 17th item, which measured attentional recovery friction by assessing how often participants found it difficult to rebuild their concentration or get back into “the zone” after their workflow was interrupted. Next, the 18th item captured self-regulatory spillover effects in non-exercise domains, tracking how often they made impulsive or less-than-ideal choices, such as in eating habits or spending, later in the day because they were tired of making structured decisions. Finally, the sub-scale concluded with the 20th item, which mapped global objective cognitive decline by quantifying how often participants experienced a feeling of “mental fog” or reduced clarity when trying to solve problems or make judgments.

#### 3.5.3 Physical activity enjoyment scale

Note on Operational Definition: Although titled under the historical scale nomenclature, the instrument utilized in this study exclusively comprises negatively worded items. Consequently, higher scores in this metric do not represent positive enjoyment; rather, they are operationalized and interpreted strictly as an inverse assessment of Physical Activity Dissatisfaction (PAD), reflecting acute affective aversion, monotony, and behavioral friction. The first dimension, Acute Affective Aversion and Monotony, systematically captured the immediate, negative emotional appraisals and sensory friction experienced by the participants during the actual performance of the physical activity sessions, which was operationalized across four specific items of the sub-scale. This dimension opened with the 21st item, which quantified immediate cognitive under-stimulation by evaluating how often participants found the required exercise routine to be boring or monotonous. The 22nd and 23rd items subsequently addressed direct emotional resistance and modality-induced irritation, tracking how frequently they felt a genuine dislike or aversion toward participating in their scheduled exercise sessions, and how often their assigned exercise mode made them feel frustrated or annoyed, respectively. Finally, the 24th item evaluated the acute loss of psychological flow and task attachment during the training bout, quantifying how often they felt completely disconnected from the exercise while doing it, feeling like they were just “going through the motions” without any sense of immersion.

The second dimension, Logistical Friction and Program Dissatisfaction, comprehensively evaluated the macro-level systemic burdens, lifestyle incompatibility, and compliance resistance surrounding the intervention protocol across the remaining six items of the instrument. This segment began by mapping the pre-behavioral and cognitive framing of the intervention, with the 25th item evaluating how often participants viewed the daily exercise sessions as a chore or an unpleasant obligation rather than a break, followed by the 26th item, which quantified immediate post-exercise mental depletion by assessing how frequently they felt unrefreshed and mentally drained after completing their exercise sessions. The 27th and 28th items targeted behavioral attrition and opportunity cost appraisals, tracking how often they thought about quitting the exercise program because they found it ungratifying, and how frequently they felt that the time spent on these exercise sessions was a waste of their valuable day, respectively. This sub-scale then concluded by evaluating global lifestyle integration friction via the 29th and 30th items, which measured how often they felt completely unsatisfied with the way exercise was integrated into their life, and how frequently they experienced an anticipatory sense of dread or mental resistance just before their scheduled exercise session was about to begin.

#### 3.5.4 Theoretical framework

The multi-dimensional architecture of the questionnaire is grounded in three complementary psychological and behavioral theories, tracking the psychological mechanism from environmental appraising to self-regulatory exhaustion and affective integration.

First, the evaluation of perceived stress is rooted in Lazarus and Folkman's Transactional Model of Stress and Coping (Lazarus and Folkman, 1984; Lazarus, 1991; Folkman and Lazarus, 1988). According to this model, stress is not merely an environmental stimulus but an evaluative process arising when contextual demands tax or exceed an individual's adaptive resources. Traditional exercise interventions typically impose predictable, block-based time blocks on a student's daily calendar. Conversely, high-frequency “exercise snacks” break the daily routine into fragmented segments, introducing multiple recurring interruptions throughout the day. By monitoring schedule disruptions, emotional strain, and perceived control deficits, the framework captures how cognitive flow fragmentation alters secondary appraisal mechanisms, revealing whether micro-interventions relieve the psychological burden of time scarcity or amplify perceived distress through chronic workflow disturbance.

Second, the structural dimension of decision fatigue and cognitive load is justified by Sweller's Cognitive Load Theory alongside Baumeister's Strength Model of Self-Regulation (Ego Depletion) (Sweller, 1988; Sweller et al., 2011; Baumeister, 2002; Vohs et al., 2008; Inzlicht and Schmeichel, 2012). Cognitive Load Theory posits that an individual's working memory capacity is strictly finite; tracking micro-logistics and frequently shifting focus back and forth between cognitively demanding tasks (e.g., studies) and physical protocol execution introduces significant task-switching costs, depleting restricted mental bandwidth. Furthermore, the Strength Model of Self-Regulation treats willpower as a limited energetic resource that is consumed by continuous executive control and behavioral monitoring. By mapping willful exhaustion, choice procrastination, and executive dysregulation later in the day, the framework evaluates whether the constant self-regulation required to adhere to an unstructured or high-frequency schedule triggers decision-making paralysis and maladaptive behaviors in unrelated personal domains.

Third, the measurement of physical activity enjoyment aligns with the Self-Determination Theory (SDT) and classical affective response frameworks in exercise psychology (Deci and Ryan, 2013; Ryan and Deci, 2000; Ekkekakis, 2003; Teixeira et al., 2012). SDT posits that behavioral adherence and long-term psychological wellbeing depend heavily on satisfying the basic psychological need for autonomy. When a physical activity intervention is perceived as monotonous, frustrating, or behaviorally mismatched with a participant's routine, it actively suppresses intrinsic motivation. This turns an active training bout into a rigid external demand, eroding task absorption and inducing flow deprivation during exercise. Furthermore, the appraisal of long-term lifestyle friction captures the tension between intervention compliance and autonomous time management, revealing whether a lack of emotional gratification drives behavioral avoidance and ultimate dropout.

### 3.6 Statistical analysis

#### 3.6.1 Baseline homogeneity and randomization efficacy

As illustrated in Table 3, the pre-intervention maximal oxygen uptake ($\dot{V}{\text{O}}_{2\text{max}}$) for the Exercise Snacks Group (ESG, *n* = 90) was 39.78 ± 8.56 mL/kg/min, whereas the Traditional Exercise Group (TEG, *n* = 90) exhibited a baseline level of 40.32 ± 7.93 mL/kg/min. The independent-samples *t*-test confirmed that this baseline variation between the two cohorts was statistically non-significant (*t*_(178)_ = −0.445, *p* = 0.657, Cohen's *d* = 0.066), with the 95% confidence interval of the difference crossing zero (−2.976 to 1.880 mL/kg/min). These results demonstrate that the random assignment protocol achieved a high degree of baseline physiological homogeneity, effectively ruling out initial aerobic capacity as a confounding variable for subsequent psychological outcomes, including perceived stress, decision fatigue, and physical activity enjoyment.

**Table 3:** Baseline homogeneity check of cardiorespiratory fitness ($\dot{V}{\text{O}}_{2\text{max}}$).

Variable	Group (Mean ±SD)		*t*	*p*
	ESG (*n* = 90)	TEG (*n* = 90)		
$\dot{V}{\text{O}}_{2\text{max}}$ (mL/kg/min)	39.78 ± 8.56	40.32 ± 7.93	−0.445	0.657

ESG, Exercise Snacks Group; TEG, Traditional Exercise Group.

Significance was set at *p* < 0.05.

#### 3.6.2 Normality test of psychometric measures

As illustrated in Table 4, the distributional architecture of the post-intervention psychometric matrices was rigorously verified. The descriptive shape parameters demonstrated that both the skewness and kurtosis values across all target variables fell safely within the universally accepted psychometric range of −1.0 to +1.0, indicating near-symmetrical shapes devoid of extreme outliers or heavy-tailed skewing.

**Table 4:** Normality distribution parameters and Shapiro–Wilk test coefficients for post-intervention scales.

Scale	Group	Skewness	Kurtosis	Shapiro–Wilk *W*	*p*
Perceived stress	ESG	0.026	−0.774	0.977	0.118
	TEG	−0.016	−0.719	0.981	0.217
Decision fatigue and cognitive load	ESG	0.009	−0.536	0.986	0.457
	TEG	0.373	−0.559	0.960	0.008
Physical activity enjoyment	ESG	0.235	−0.314	0.983	0.294
	TEG	−0.037	0.116	0.978	0.123

Significance threshold was set at *p* < 0.05.

The Shapiro–Wilk (*W*) test further substantiated the mathematical assumption of normality for the majority of the sub-scales. Specifically, the continuous scores for Perceived Stress (ESG: *W* = 0.977, *p* = 0.118; TEG: *W* = 0.981, *p* = 0.217) and Physical Activity Enjoyment (ESG: *W* = 0.983, *p* = 0.294; TEG: *W* = 0.978, *p* = 0.123) demonstrated an ideal alignment with a normal distribution model (*p*>0.05). Within the Decision Fatigue and Cognitive Load domain, the ESG cohort achieved robust compliance with normality (*W* = 0.986, *p* = 0.457). Although the corresponding TEG cohort within this domain exhibited a minor mathematical deviation from strict normality (*W* = 0.960, *p* = 0.008), its absolute skewness (0.373) and kurtosis (−0.559) were well within standard linear thresholds. Given the adequate statistical power yielded by the sample size (*n* = 90 per group) and the natural resilience of parametric analysis of variance models against slight non-normality, the baseline distribution was deemed fully eligible for subsequent robust parametric comparisons.

### 3.7 Inter-variable correlation

#### 3.7.1 Group-specific correlation analysis and psychometric design validation

To fully evaluate the internal psychological architecture under different exercise paradigms and validate the empirical independence of our metrics, Pearson correlation matrices were initially constructed within the Traditional Exercise Group (TEG) and the Exercise Snacks Group (ESG) independently.

As illustrated in Figure 4, when analyzed within their respective cohorts, the variables displayed surprisingly low to negligible linear couplings. Within the ESG cohort, the correlation coefficients across all variable pairs neared mathematical zero, ranging from −0.030 to 0.087, indicating complete operational independence. In parallel, the TEG cohort retained a marginally higher but still weak correlation topology, with coefficients spanning from −0.047 to 0.218.

**Figure 4:**
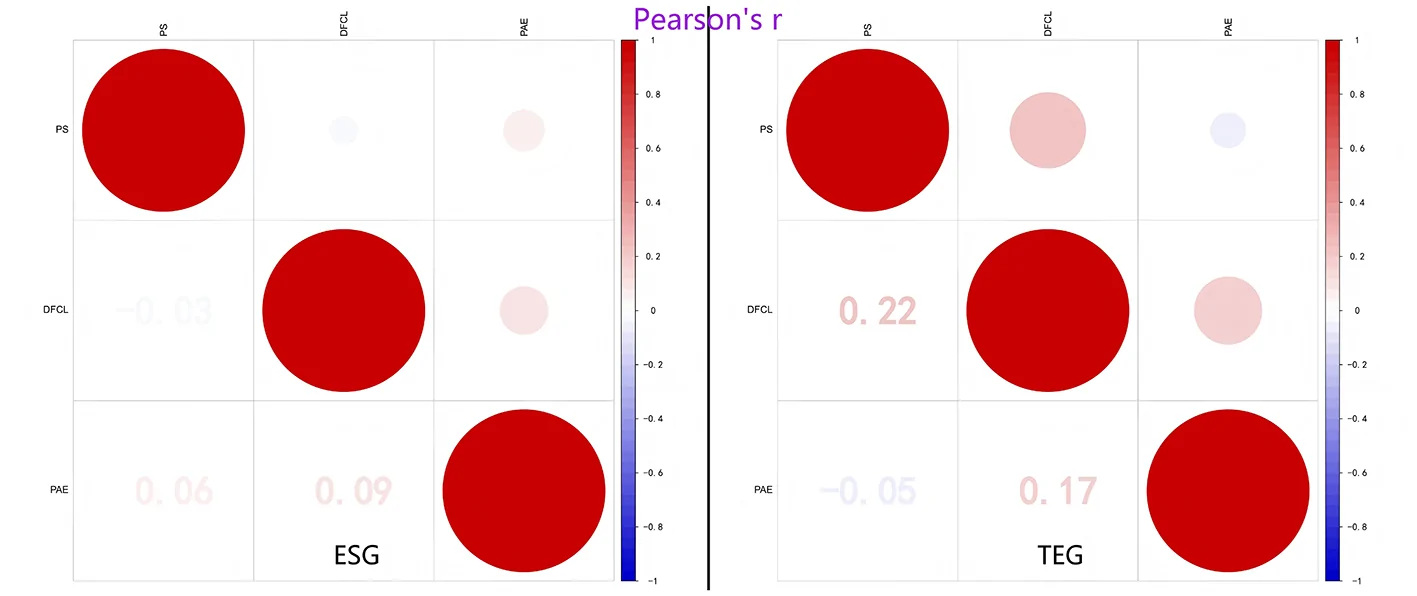
Comparative analysis of psychometric correlation structures by intervention modality.

Critically, this intra-group decoupling highlights the outstanding success of our psychometric indicator design, specifically demonstrating excellent discriminant validity and freedom from common method bias (CMB). In behavior-tracking studies, a frequent flaw is that participants' overlapping psychological states pollute separate questionnaires, generating artificially inflated correlations within a single group. However, the near-zero and weak correlation coefficients observed here prove that Perceived Stress (PS), Decision Fatigue (DFCL), and Physical Activity Enjoyment (PAE) were successfully isolated by our measurement scales as distinct, non-redundant cognitive and affective dimensions. Participants evaluated their cognitive fatigue, environmental stress, and emotional enjoyment as separate entities rather than a single confounded affective state, fundamentally establishing the scientific accuracy and construct validity of the selected psychometric battery.

## 4 Discussion

### 4.1 Implications in light of prior research

While existing literature extensively documents the metabolic and physiological advantages of fragmented exercise, our psychometric evaluations reveal critical divergences regarding the psychological burden of high-frequency micro-scheduling (Babir et al., 2025; Alexe et al., 2025; Stork et al., 2023; Lee and Jo, 2026; Fyfe et al., 2022). Conventional paradigms generally assume that breaking down exercise into shorter bouts enhances feasibility and lowers barriers; however, the Exercise Snacks paradigm (ESG) in our study introduced significant behavioral friction and cognitive strain. This is evidenced by significantly elevated Perceived Stress (PS) and a nearly threefold increase in attrition compared to the Traditional Exercise Group (TEG; 15.56 vs. 5.56%). Drawing upon Lazarus and Folkman's Transactional Model of Stress and Coping, the repeated daily task-switching required by ESG appears to disrupt psychological equilibrium, a nuance overlooked in volume-equated continuous protocols. Furthermore, these findings extend Sweller's Cognitive Load Theory and the Self-Determination Theory frameworks by demonstrating that the frequent executive interruptions of “exercise snacks” impose hidden mental costs. Consequently, while fragmented protocols successfully circumvent time constraints, their psychological toll and impact on intrinsic motivation must be carefully weighed against their physiological benefits.

### 4.2 Sample homogeneity and implications for external validity

While the strict inclusion criteria ensured baseline physiological homogeneity, the exclusive reliance on university students (aged 18–27) may limit the generalizability of these findings to older populations or individuals with higher vocational stress levels. Future investigations should incorporate diverse cohorts to validate whether the observed “behavioral friction” of the Exercise Snacks Group (ESG) persists across different socio-occupational contexts.

### 4.3 Psychometric rigor and the mitigation of response bias

While self-reported psychological assessment is susceptible to response bias, several measures were taken to reinforce the psychometric rigor of this study. By adopting standardized psychological scales and maintaining strict anonymity, we minimized the potential for systematic reporting errors. The observed divergence in psychological impact between the experimental groups is robust, given the careful calibration of the exercise protocols and the longitudinal nature of the data collection. However, acknowledging the subjective nature of these metrics, we view these results as a foundational exploration of the “exercise snack” phenomenon, which should be complemented by multi-modal assessment techniques in subsequent studies to further delineate the relationship between exercise fragmentation and cognitive appraisal.

### 4.4 Temporal dynamics and long-term psychological adaptation

The 4-week intervention period, while sufficient to capture the initial psychometric response to exercise fragmentation, may not fully elucidate the trajectory of long-term psychological adaptation. Critics might argue that the observed increase in perceived stress and decision fatigue could diminish as participants habituate to the frequent, short-duration exercise bouts. We acknowledge that the current study focuses on the early-stage “behavioral friction” phase; therefore, these findings should be interpreted as a baseline for understanding immediate cognitive load. Future longitudinal studies extending beyond 4 weeks are warranted to determine whether psychological resilience builds over time, potentially mitigating the initial strain associated with high-frequency exercise protocols.

### 4.5 Methodological robustness of the participant replacement protocol

A critical methodological vulnerability in longitudinal exercise trials involves data integrity following participant attrition and subsequent protocol replacement. While our pre-screened standby recruitment pool successfully maintained the nominal sample size (*N* = 90 per group) and statistical power, we explicitly acknowledge that replacing non-adherent subjects introduces selective survivorship artifacts. Specifically, substituting dropouts with compliant baseline-matched volunteers inherently filters out extreme behavioral friction, thereby artificially suppressing true population-level attrition rates and deflating the apparent variance in post-test psychometric distributions. Consequently, while this dynamic replacement protocol preserves statistical power for comparative analyses, the resulting psychometric profiles reflect an idealized compliant subset rather than an unmitigated intention-to-treat reality, a limitation that must be accounted for when interpreting real-world generalizability.

### 4.6 Synthesis of evidence and future predictive modeling

In light of the psychological insights derived from this study, we propose that future research transition toward more sophisticated behavioral tracking frameworks grounded directly in exercise adherence dynamics. Rather than relying solely on post-intervention evaluations, subsequent investigations should incorporate real-time ecological momentary assessment (EMA) and wearable sensor technologies to continuously monitor acute fluctuations in perceived stress and cognitive load during fragmented exercise protocols (Zhang and Liu, 2025; Yi et al., 2026). By capturing micro-longitudinal behavioral shifts, researchers can map the temporal decay of intrinsic motivation and proactively identify the critical tipping points of behavioral friction before protocol abandonment occurs. This domain-specific direction directly extends our findings, offering a robust pathway to design adaptive, personalized exercise snack prescriptions that optimize psychological wellbeing and long-term compliance in general populations.

### 4.7 Methodological rationale for the low-to-moderate exercise intensity

A potential critique of our experimental design is the selection of 40% of $\dot{V}O_{2max}$ as the standardized intervention intensity, which represents the lower boundary of moderate-intensity exercise and imposes minimal physiological strain compared to high-intensity interval training (HIIT) protocols commonly found in the literature. However, this intensity was a deliberate methodological choice rather than a physiological oversight. The primary objective of this investigation was to isolate and evaluate behavioral, logistical, and cognitive friction—such as task-switching costs, scheduling disruptions, and self-regulatory depletion—rather than to maximize cardiovascular or metabolic overload. In cohorts of previously sedentary young adults, high-intensity protocols frequently induce severe physical discomfort, musculoskeletal fatigue, and delayed-onset muscle soreness, which would introduce confounding physical distress into psychometric appraisals of perceived stress and enjoyment. By standardizing the physical workload at a manageable 40% of $\dot{V}O_{2max}$, we effectively neutralized physiological strain as a confounding variable, ensuring that the observed elevations in Perceived Stress (PS) and Decision Fatigue and Cognitive Load (DFCL) within the Exercise Snacks Group (ESG) stem purely from the high-frequency structural demands and micro-scheduling friction of the protocol rather than physical exhaustion.

### 4.8 Methodological limitation regarding baseline psychological assessments

A notable limitation of the current study is the absence of pre-intervention baseline psychological assessments for Perceived Stress (PS), Decision Fatigue and Cognitive Load (DFCL), and Physical Activity Enjoyment (PAE). While rigorous baseline homogeneity was successfully established for physical fitness via maximal oxygen uptake ($\dot{V}O_{2max}$) testing, we did not measure initial psychological profiles prior to the 4-week intervention. Consequently, we cannot entirely rule out the possibility of pre-existing baseline psychological differences between the Exercise Snacks Group (ESG) and the Traditional Exercise Group (TEG). Although random assignment via a computer-generated block randomization method theoretically mitigates allocation bias, future longitudinal investigations should incorporate pre-intervention psychometric baseline mapping to definitively control for pre-existing individual variances and strengthen causal inferences.

## 5 Conclusion

This study provides a comprehensive comparative evaluation of the psychological and behavioral impacts of two distinct exercise modalities—Exercise Snacks Group (ESG) and Traditional Exercise Group (TEG) —within a controlled 4-week longitudinal framework. Our findings reveal three primary insights regarding the implementation of fragmented vs. continuous exercise protocols.

First, while both modalities utilize identical total volume and intensity (40% $\dot{V}O_{2max}$), they elicit fundamentally divergent psychometric profiles based on our study-specific assessment tools. Specifically, when evaluated via the current questionnaire matrix, the ESG protocol is associated with higher scores in items reflecting perceived schedule disruption and decision strain rather than broad, generalized psychological constructs. In accordance with the lack of external psychometric validation beyond this trial, these conclusions are strictly limited to the participants' specific item-level responses within this experimental context. This divergence highlights the non-negligible cognitive and logistical costs inherent in “snacking” exercise, which may act as a significant barrier to long-term routine integration for individuals with demanding daily schedules.

Second, the attrition rates observed in this study provide critical evidence of real-world viability. The three-fold increase in dropout rates within the ESG cohort (15.56%) compared to the TEG cohort (5.56%) suggests that the task-switching friction and repetitive behavioral engagement required by high-frequency fragmented exercise create a psychological disruption that complicates consistent adherence. This underscores the necessity of considering “behavioral friction” as a key design factor in exercise prescription for general populations.

Finally, the shift from intra-group independence to aggregate parametric convergence—as evidenced by the surge in correlation coefficients upon pooling the data—suggests that the physiological and psychological responses to exercise intensity are highly dependent on the intervention structure. While fragmented exercise provides an alternative modality to bypass the barrier of prolonged single-session duration, its potential to induce unintended cognitive and emotional strain requires careful consideration. In conclusion, while Exercise Snacks offer a schedule-flexible approach by distributing physical activity, practitioners and researchers must balance the physical benefits against the psychological demands of the protocol to ensure sustainable and well-tolerated exercise adherence among diverse participant populations.

## Data Availability

The raw data supporting the conclusions of this article will be made available by the authors, without undue reservation.
