# Supplementary material for: Psychological impacts of exercise snacks vs. traditional exercise: a comparative study
Source: Front Psychol. 2026 Oct 8;17:1917848. doi: 10.3389/fpsyg.2026.1917848 (PMC13686887; doi:10.3389/fpsyg.2026.1917848)
Supplement: Supplementary file 1 [file Data_Sheet_1.pdf]

Response Options (5-point Likert Scale): 1 = Never, 2 = Almost Never, 3 = Sometimes, 4 = Fairly Often, 5 = Very Often.

Perceived Stress

1. In the past month, how often have you felt upset because of unexpected changes in your daily schedule, such as your routine being interrupted by the exercise intervention?

2. In the past month, how often have you felt a frustrating lack of control over the important things in your life?

3. In the past month, how often have you felt generally nervous, stressed, or "on edge"?

4. In the past month, how often have you felt overwhelmed by the challenge of balancing your work/studies with your daily personal responsibilities?

5. In the past month, how often have you felt that the difficulties and demands accumulating in your life were too heavy for you to handle?

6. In the past month, how often have you found it difficult to mentally unwind or relax, even when you had free time?

7. In the past month, how often have you felt emotionally drained or easily irritated by minor hassles in your daily routine?

8. In the past month, how often have you experienced a persistent sense of time urgency, feeling that you were constantly racing against the clock?

9. In the past month, how often have you felt angry or frustrated because you felt inefficient or unproductive due to frequent daily interruptions?

10. In the past month, how often have you felt that you lacked the mental bandwidth or social support needed to cope with your daily stressors?

Decision Fatigue and Cognitive Load

11. In the past month, how often have you felt that planning and scheduling your daily activities (including fitting in your exercise sessions) required significant mental effort?

12. In the past month, how often have you felt mentally exhausted from

constantly shifting your focus back and forth between your work/studies and your exercise routine?

13. In the past month, how often have you found yourself delaying or procrastinating on making simple decisions because you felt mentally drained?

14. In the past month, how often have you felt that your willpower was completely drained by the end of the day due to the constant need to self-regulate and stick to your schedule?

15. In the past month, how often have you felt that your brain was "overloaded" with too many small details and tasks to keep track of throughout the day?

16. In the past month, how often have you felt a strong desire to avoid making choices altogether, preferring to let others decide or let things slide?

17. In the past month, how often have you found it difficult to rebuild your concentration or get back into "the zone" after your workflow was interrupted?

18. In the past month, how often have you made impulsive or less-than-ideal choices (e.g., in eating habits or spending) later in the day because you were tired of making structured decisions?

19. In the past month, how often have you felt that your mental bandwidth was so restricted that you could only focus on immediate deadlines, losing sight of the bigger picture?

20. In the past month, how often have you experienced a feeling of "mental fog" or reduced clarity when trying to solve problems or make judgments?

Physical Activity Enjoyment

21. In the past month, how often have you found the required exercise routine to be boring or monotonous?

22. In the past month, how often have you felt a genuine dislike or aversion toward participating in your scheduled exercise sessions?

23. In the past month, how often has your assigned exercise mode made you feel frustrated or annoyed?

24. In the past month, how often have you felt completely disconnected from

the exercise while doing it, feeling like you were just "going through the motions" without any sense of immersion?

25. In the past month, how often have you viewed the daily exercise sessions as a chore or an unpleasant obligation rather than a break?

26. In the past month, how often have you felt unrefreshed and mentally drained after completing your exercise sessions?

27. In the past month, how often have you thought about quitting the exercise program because you found it ungratifying?

28. In the past month, how often have you felt that the time spent on these exercise sessions was a waste of your valuable day?

29. In the past month, how often have you felt completely unsatisfied with the way exercise was integrated into your life?

30. In the past month, how often have you felt a sense of dread or mental resistance just before your scheduled exercise session was about to begin?
